# Real-world outcomes of a multidisciplinary rehabilitation programme for adults with long COVID: a retrospective cohort study

**DOI:** 10.1186/s12889-026-29023-3

**Published:** 2026-08-13

**Authors:** Lanre Peter Daodu, Kim Nottingham, Yogini Raste, Judith E. Allgrove, Francesca I. F. Arrigoni, Reem Kayyali

**Affiliations:** 1 Faculty of Health, Sciences, Social Care and Education, Kingston University, London, UK; 2 Respiratory Physiotherapy Unit, Croydon University Hospital, London, UK; 3 Chest/Respiratory Unit, Internal Medicine, Croydon University Hospital, London, UK; 4 School of Allied Health and Exercise Sciences, Faculty of Health, Environment and Medical Sciences, Bournemouth University, Bournemouth, BH12 5BB UK

**Keywords:** Post-acute COVID-19 syndrome [MeSH], Rehabilitation [MeSH], Dyspnoea [MeSH], Functional status [MeSH], Physical fitness [MeSH], Exercise tolerance [MeSH]

## Abstract

**Background:**

Long COVID imposes a substantial and ongoing burden, with persistent symptoms affecting daily functioning and quality of life. Multidisciplinary rehabilitation has been widely implemented in clinical practice; however, evidence describing real-world patient outcomes and service delivery remains limited. This study aimed to describe clinical and patient-reported outcomes among adults participating in a structured multidisciplinary rehabilitation programme for Long COVID, and to explore the association between referral-to-assessment time and changes in outcomes.

**Methods:**

This retrospective cohort study analysed routinely collected clinical data from adult patients who completed a structured multidisciplinary rehabilitation programme at the Long COVID rehabilitation centre in a university hospital in South London (January 2021–December 2023). Seven validated outcome instruments were administered at baseline and discharge: the Work and Social Adjustment Scale (WSAS), Fatigue Severity Scale (FSS), MRC Dyspnoea Scale, Patient Health Questionnaire-2 (PHQ-2), Generalised Anxiety Disorder-2 (GAD-2), EQ-5D-5 L, and 1-minute Sit-to-Stand test (STS-1). Pre-to-post changes were assessed using paired t-tests and Wilcoxon signed-rank tests; the proportion of patients achieving minimal clinically important differences (MCIDs) was determined for each measure. The association between referral-to-assessment time and outcome change was examined using linear correlation and restricted cubic spline (RCS) modelling.

**Results:**

A total of 170 participants were included. Statistically significant improvements were observed across all primary domains after adjustment for multiple comparisons, except self-care and pain/discomfort. The largest effect was recorded for physical endurance (STS-1: *r* = 0.590, *p* < 0.001), followed by breathlessness (MRC: *r* = 0.491), fatigue (FSS: *r* = 0.382), and functional impairment (WSAS: d = − 0.323). MCID thresholds were achieved by 63.5% of patients on the STS-1, 48.8% for breathlessness, and 45.9% for depression. No significant linear association was identified between referral delay and outcome change. Non-linear therapeutic windows were identified in functional and psychological domains using RCS modelling. Patients assessed 100–150 days after referral showed the greatest improvement, with benefits declining thereafter.

**Conclusion:**

Participants demonstrated improvements in clinical and patient-reported outcomes over time during engagement in a multidisciplinary rehabilitation programme for Long COVID. While causal attribution is limited by the observational design, these findings provide real-world evidence on patient outcomes and highlight the potential importance of timely access to rehabilitation services. Further prospective and controlled studies are needed to evaluate effectiveness and optimise care pathways.

**Supplementary Information:**

The online version contains supplementary material available at https://doi.org/10.1186/s12889-026-29023-3.

## Introduction

Long COVID is defined as an infection-associated chronic condition that occurs after severe acute respiratory syndrome coronavirus 2 (SARS-CoV-2) infection and is present for at least 3 months as a continuous, relapsing and remitting, or progressive disease state that affects one or more organ systems [1]. Epidemiological data indicate that the global pooled prevalence of Long COVID is approximately 36% [2], with a cumulative incidence of around 400 million individuals worldwide as of 2024 [3]. Beyond the clinical burden, the condition carries a staggering socio-economic cost, with an estimated annual global economic impact of $1 trillion, equivalent to roughly 1% of the global economy [3]. The condition impacts multiple organ systems, causing fatigue, breathlessness, cognitive issues, and psychological distress, which can significantly impair daily functioning and quality of life (QoL) [4, 5]. Many individuals are unable to work or participate socially, and healthcare systems face increased demand for specialised services [6]. In the United Kingdom (UK), the Office for National Statistics (ONS) reported that as of March 2024, approximately 2.0 million people in England and Scotland (3.3% of the population) were experiencing Long COVID symptoms [7], with a significant proportion reporting substantial limitations in their daily activities. The complexity and prevalence of Long COVID highlight the need for coordinated evidence-based rehabilitation strategies.

Multidisciplinary rehabilitation — integrating physiotherapy, occupational therapy, psychological support, and self-management education — has been adopted as a cornerstone of Long COVID care across many health systems, guided by national and international clinical frameworks [8]. Emerging evidence suggests that graded exercise therapy (GET), respiratory physiotherapy, and pacing strategies may improve physical capacity, reduce fatigue burden, and alleviate breathlessness in Long COVID cohorts [9, 10]. However, the existing literature is constrained by several methodological limitations: many existing studies are relatively small and often lack standardised outcome reporting frameworks; studies are predominantly small, limiting comparability and the strength of evidence available to guide practice [11]. Furthermore, most investigations have not employed validated, multidomain outcome instruments — such as the Work and Social Adjustment Scale (WSAS), Fatigue Severity Scale (FSS), MRC Dyspnoea Scale, Patient Health Questionnaire-2 (PHQ-2), Generalised Anxiety Disorder-2 (GAD-2), EQ-5D-5 L, the EQ Visual Analogue Scale (EQ-VAS), or 1-minute Sit-to-Stand test (STS-1)— applied systematically at both programme entry and discharge, thereby limiting the rigour of pre-to-post comparisons and clinical inference [12, 13].

A critical but poorly characterised aspect of Long COVID rehabilitation care is the potential influence of referral-to-treatment delay on clinical outcomes. In established rehabilitation medicine, the principle of early intervention is well recognised: delayed access to rehabilitation following acute illness or injury is associated with greater functional decline, psychological deterioration, and reduced responsiveness to intervention [14]. Yet, in the context of Long COVID, no consensus exists on the optimal timing of rehabilitation initiation, and the relationship between the time from referral to first assessment and subsequent functional and psychological gains has not been systematically examined [15]. This gap is clinically important; given the variability in referral pathways, triage practices, and service capacity across health systems, understanding whether delayed access attenuates rehabilitation benefit — or whether therapeutic gains remain achievable irrespective of delay — has direct implications for service design, patient prioritisation, and resource allocation [6, 16]. Moreover, the real-world effectiveness of structured Long COVID rehabilitation, evaluated using validated instruments across functional, physical, psychological, and quality-of-life domains simultaneously, remains insufficiently characterised in large, routinely collected clinical datasets [11].

To address these evidence gaps, we analysed data from adult Long COVID patients who underwent and completed the Long COVID rehabilitation programme. We aimed to: (i) quantify changes following rehabilitation in functional status, physical performance, psychological well-being, and health-related quality of life using validated outcome measures; (ii) determine the proportion of participants achieving the minimal clinically important differences (MCID) for each outcome measure; and (iii) examine whether referral-to-assessment time is associated with the magnitude of improvement in rehabilitation outcomes.

## Methods

### Study design

This study was a retrospective cohort analysis of routinely collected clinical data from patients with Long COVID. The participants underwent structured multidisciplinary rehabilitation at the Long COVID rehabilitation centre in the university hospital in South London between January 2021 and December 2023.

### Study setting and eligibility criteria

This hospital-based study was conducted at a secondary healthcare institution in South London. The structured multidisciplinary rehabilitation programme typically spanned 12 weeks and was tailored to individual clinical needs. Delivered via onsite and virtual formats, the intervention integrated fatigue management education, psychological support, and, where appropriate, group-based graded exercise or movement therapy to address deconditioning while monitoring for post-exertional malaise (PEM).

The study cohort comprised participants (≥ 18 years) diagnosed with Long COVID who were referred from any of the eight Primary Care Networks and healthcare service clusters operating within the London Borough of Croydon. The diagnosis of Long COVID was established through a multidisciplinary team clinical triage in accordance with NICE guideline [NG188] [8]. This process involved a face-to-face or virtual assessment by a senior clinician (physician or specialist physiotherapist) who utilised the Newcastle screening tool [17] to confirm a history of acute SARS-CoV-2 infection via polymerase chain reaction (PCR), lateral flow device (LFD). Clinical confirmation required the exclusion of alternative underlying pathologies—such as cardiac or pulmonary complications—and the presence of a symptom profile that persisted for at least 12 weeks, ensuring that the cohort met the criteria for Post-COVID-19 Syndrome.

To be included in the final analysis, participants must have completed the full 12-week structured rehabilitation programme with documented baseline and endline assessments. Participants were excluded if they lacked documented referral outcomes, were deemed clinically unsuitable for rehabilitation due to active medical contraindications, or if the study team was unable to establish contact. Further exclusion criteria included declining participation, spontaneous recovery prior to the intervention, failure to complete the programme, non-attendance at the final reassessment, or death during the study period.

### Data source, collection and variables

All data were extracted from the long COVID rehabilitation centre patient management files using a structured Excel sheet. Variables extracted included demographic (age, sex and ethnicity), referral sources, referral-to-assessment time (calculated as the number of days between the date of referral and the date of the first rehabilitation session), functional outcomes such as WSAS and STS-1, symptoms severity such as FSS and MRC dyspnoea scale, psychological outcomes such as PHQ-2 and GAD-2, QoL parameters: EQ-5D-5L (domains) and EQ-5D visual analogue scale (VAS). The description and scoring of each variable/instrument are in Supplementary File 1.

### Study outcomes and validation

For each outcome measure, a change score (Δ) was computed as:

1 $$\Delta=\text{Endline Score}-\text{Baseline Score}$$

Minimal clinically important differences (MCIDs) were defined based on published literature: the WSAS assessed the impact of symptoms across five life domains (work, home management, social leisure, private leisure, and relationships). Scores range from 0 to 40, with higher scores indicating greater functional impairment [18]. The FSS consists of 9 items, rated on a 7-point Likert scale (total score 1–7), with higher scores indicating greater fatigue impact on daily living [19]. Breathlessness was assessed using the MRC Dyspnoea Scale [20], a 5-point scale in which Grade 1 indicates minimal breathlessness and Grade 5 maximal disability. Depressive and anxiety symptoms were screened using the PHQ-2 [21] and the GAD-2 [22], respectively. Both instruments range from 0 to 6, with higher scores indicating increased symptom frequency and severity over the preceding 2 weeks. The EQ-5D-5 L [23] assessed HRQoL across five domains (mobility, self-care, usual activities, pain/discomfort, and anxiety/depression) on a 1–5 scale. Additionally, the EQ-VAS [24] assessed self-rated health on a 0–100 scale, where 100 represents the “best imaginable health.” Physical endurance was measured using the STS-1 test [25], which measures the number of repetitions completed in 60 s; higher counts reflect improved lower-limb strength and exercise capacity.

### Statistical analysis

All statistical analyses were performed using *R* (version 4.3.3). Missing data, which occurred randomly and affected fewer than 20% of cases, were addressed using Multiple Imputation by Chained Equations (MICE). To ensure the stability of the estimates, 20 imputed datasets were generated over 20 iterations. The predictor matrix was optimised using the quickpred function, selecting predictors with a minimum correlation of 0.1 and at least 25% usable cases. Administrative date variables (referral, assessment, and completion dates) were excluded from the imputation model to maintain data integrity. Predictive mean matching was employed for all continuous variables, and the final results were pooled according to Rubin’s rules.

### Descriptive statistics

Continuous variables were assessed for normality using the Shapiro–Wilk test. Normally distributed variables were reported as means with standard deviations, while non-parametric variables were presented as medians with interquartile ranges. Categorical variables were summarised using frequencies and percentages.

### Change in outcomes in the rehabilitation

Paired-samples t-tests were used to assess outcome changes from baseline to discharge for outcomes that followed a normal distribution, while the Wilcoxon signed-rank test was applied to variables that were not normally distributed. Effect sizes were calculated using Cohen’s *d* for normally distributed paired data to assess the magnitude of change and rank-biserial correlation (r_rb_) for non-parametric comparisons. Benchmarks for Cohen’s d (used with t-tests and mean differences): small effect: d ≈ 0.20; moderate effect: d ≈ 0.50; and large effect: d ≈ 0.80 or greater. For correlation coefficients (r), often reported in Wilcoxon signed-rank tests, correlations, and regressions: small effect: *r* ≈ 0.10; moderate effect: *r* ≈ 0.30; and large effect: *r* ≈ 0.50 or greater. The p-values were adjusted using the Benjamini–Hochberg false discovery rate (FDR) method to control for multiple comparisons, with significance set at *p* < 0.005.

Correlation analyses were conducted to examine the association among changes in functional, psychological, and QoL outcomes. Change scores were calculated by subtracting baseline from endline values; negative scores indicate improvement for measures in which higher scores indicate greater impairment. Pearson’s product–moment correlation coefficients (r) were computed to assess the linear association between change scores across domains. This approach provided a standardised effect-size metric, allowing the interpretation of the magnitude and direction of associations relative to usual benchmarks.

Specifically, r values were categorised as 0.10 (small), 0.30 (moderate), and 0.50 (large). Correlations’ significance was assessed with two-tailed tests; p-values < 0.05 were significant. FDR correction was also used for multiple comparisons. The FDR is a statistical method used in multiple hypothesis testing to control the expected proportion of false positives (Type I errors) among all rejected hypotheses (discoveries) [26, 27].

### Referral-to-assessment time association with changes in outcomes

To assess whether referral-to-assessment time was associated with changes in outcomes, univariable associations were first examined using Pearson correlation coefficients for normally distributed variables and Spearman’s rank correlation for skewed variables. When non-linearity was suspected between referral time and outcomes, RCS were used to model the relationship, following Harrell’s approach [28]. Model assumptions, including residuals’ normality, linearity, homoscedasticity, and multicollinearity (variance inflation factor < 5), were evaluated for robustness.

## Results

### Participants flow

**Fig. 1:**
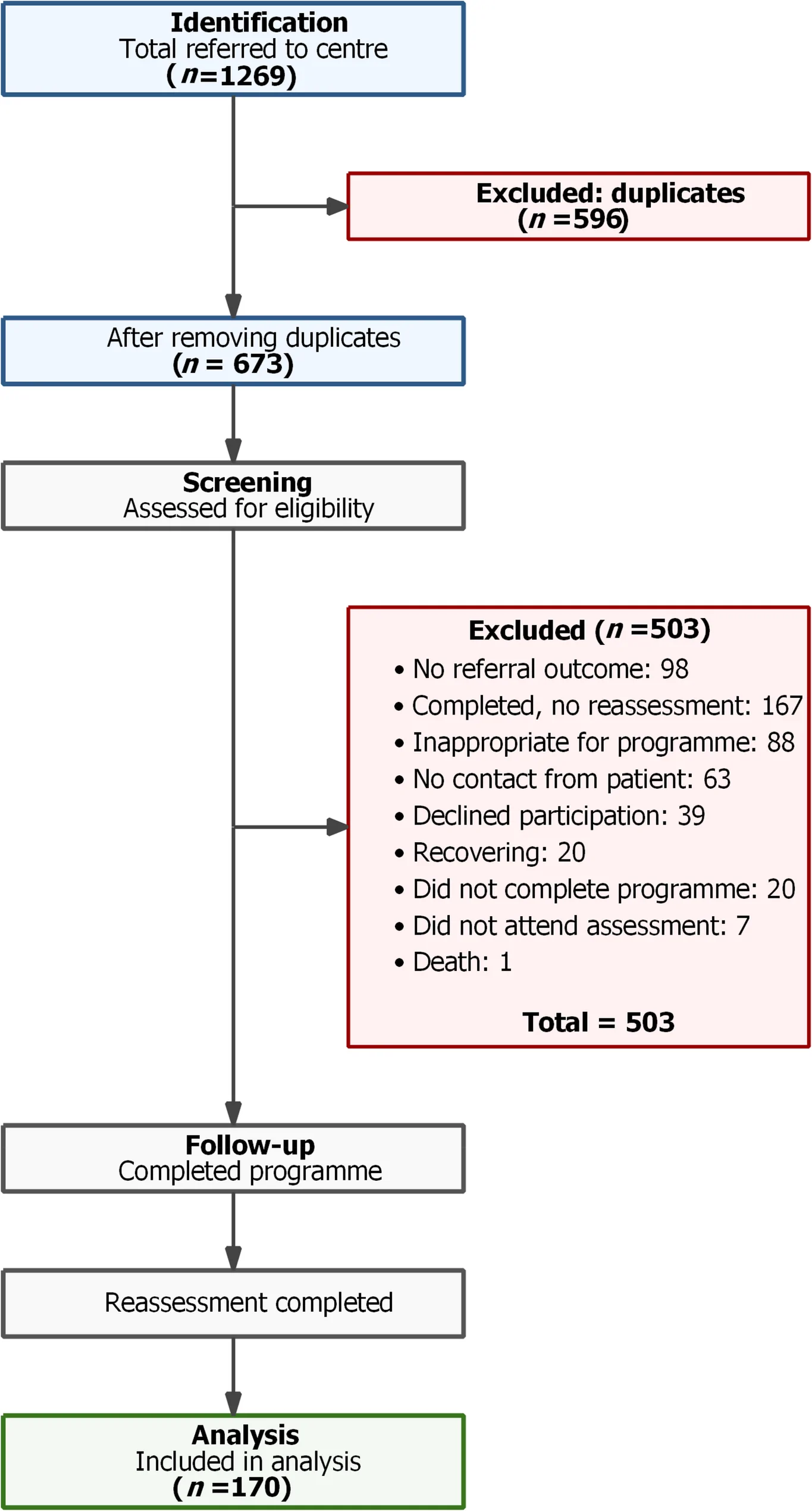
Flow chart of participants’ referral, exclusion, and rehabilitation programme outcomes during the study period

The participant selection process is summarised in Fig. 1. A total of 1269 individuals were referred to the centre during the study period. After excluding duplicates (*n* = 596), 673 distinct patients were identified and assessed for eligibility. Of these, 503 individuals were excluded for the following reasons: lack of a documented referral outcome (*n* = 98); completion of the programme without a final reassessment (*n* = 167); deemed inappropriate for the programme during triage (*n* = 88); no contact established (*n* = 63); declined participation (*n* = 39); recovering (*n* = 20); failure to complete the programme (*n* = 20); non-attendance at the assessment (*n* = 7); and death (*n* = 1). Consequently, a final cohort of 170 participants who completed the programme and provided both baseline and post-programme outcome data was included in the final analysis.

### Referral patterns and demographic profile

Referral volume peaked at 50.7% in 2021, then declined sharply through 2022 and 2023 (Supplementary File 2). Stratification by age group showed that the 55–64-year-old group consistently had the highest number of referrals over three years, indicating that middle-aged and older adults primarily used the service. Female patients outnumbered males, and referrals declined for both over time. White patients were the largest referral group each year, about 42.6% overall, up from 40.6% in 2021 to 50.9% in 2023. Black patients accounted for 20.3%, Asian patients for 16.1%, and other ethnicities for 21.0%.

### Baseline and endline outcome distributions

**Fig. 2:**
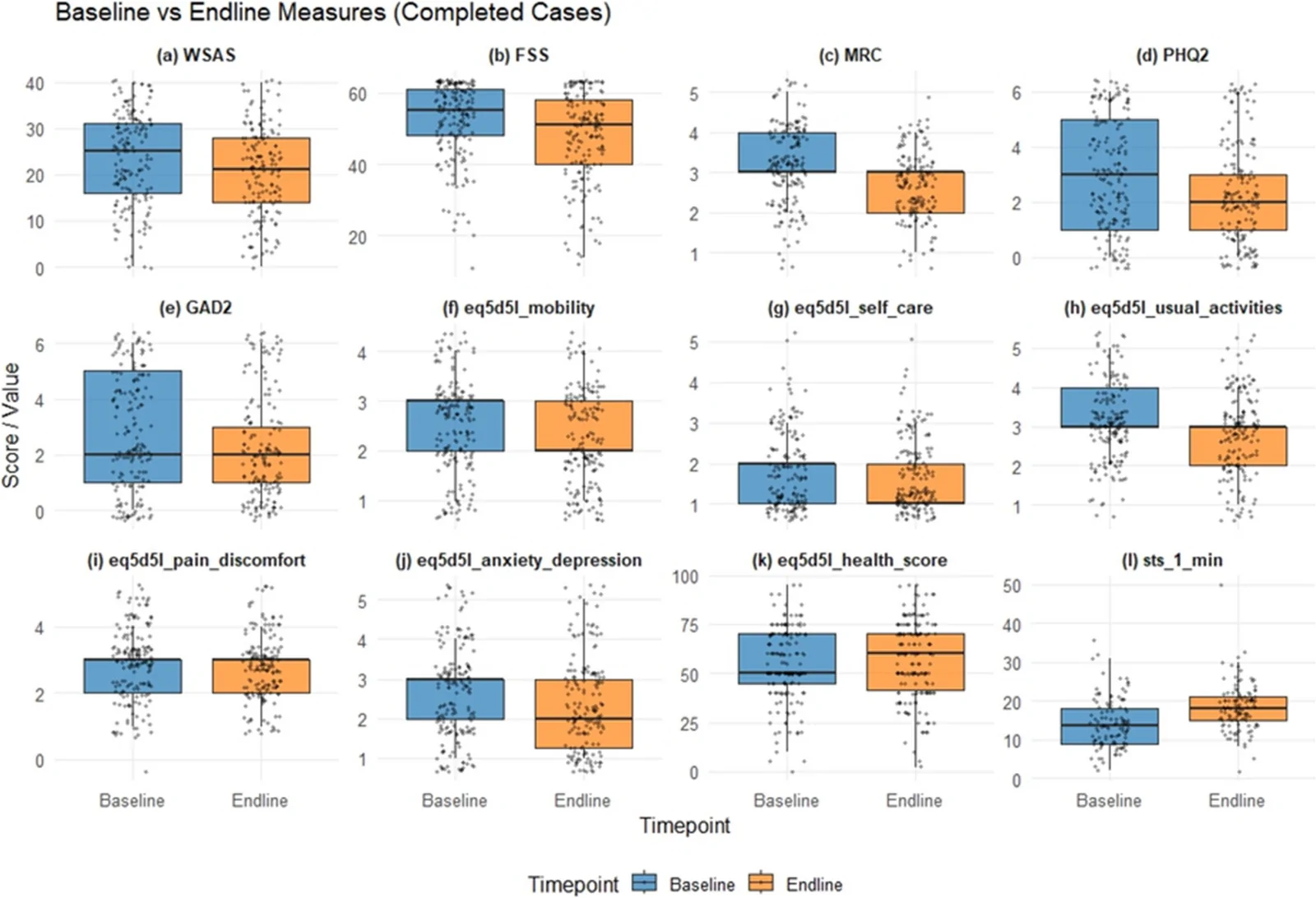
Distribution of baseline and endline outcome measures among participants who completed rehabilitation. Paired boxplots depict changes in clinical and functional scores from admission (Baseline, blue) to discharge (Endline, orange). The horizontal line within each box indicates the median; box edges represent the interquartile range (IQR, 25th–75th percentiles); whiskers extend to the most extreme values within 1.5×IQR. Individual data points are overlaid to illustrate sample variability

Figure 2 presents paired boxplots with overlaid individual data points for all outcome measures, enabling direct comparison of central tendency and dispersion between programme entry (baseline) and discharge (endline). Functional impairment (WSAS; panel a) showed a downward shift in both the median and interquartile range, indicating improved social and occupational functioning. Fatigue severity (FSS; panel b) showed a clear reduction in the median, interquartile range, and high-end outliers, reflecting a clinically significant reduction in fatigue burden, although inter-individual variability persisted. Breathlessness (MRC dyspnoea scale; panel c) decreased from baseline to endline, consistent with the functional gains observed on the sit-to-stand test.

Depressive symptoms (PHQ-2; panel d) and anxiety (GAD-2; panel e) both shifted towards the lower end of their respective scales at endline, with GAD-2 values clustering in the lower range, indicating consistent mental health improvement across the cohort. For health-related QoL (HRQoL), all five EQ-5D-5 L domains — mobility (panel f), self-care (panel g), usual activities (panel h), pain/discomfort (panel i), and anxiety/depression (panel j) — showed lower severity scores at endline, suggesting broader functional and symptomatic recovery. The EQ-5D-5 L health score visual analogue scale (VAS; panel k) showed an upward shift in the median, reflecting improved self-rated health. Objective physical capacity, assessed by the STS test (STS-1; panel l), showed a substantial increase in repetitions at endline, indicating marked recovery in lower-limb endurance and exercise capacity.

### Pre-to-post change analysis

Significant improvements were statistically observed across functional, physical, psychological, and HRQoL domains after rehabilitation (Table 1). The largest effect was observed for physical endurance: the STS-1 test showed a large effect size (*r* = 0.590, adjusted *p* < 0.001), indicating a substantial increase in lower-limb functional capacity. Breathlessness (MRC dyspnoea scale; *r* = 0.491, adjusted *p* < 0.001) and fatigue (FSS; *r* = 0.382, adjusted *p* < 0.001) both improved with medium-to-large effect sizes, confirming that the programme showed significant observed improvements in two of the most prevalent and disabling symptoms of Long COVID. Functional impairment (WSAS) improved significantly on a paired t-test (d = − 0.323, adjusted *p* < 0.001), reflecting significant gains in occupational and social functioning. Psychological outcomes improved significantly, albeit with moderate effect sizes: depressive symptoms (PHQ-2; *r* = 0.321, adjusted *p* < 0.001) and anxiety (GAD-2; *r* = 0.314, adjusted *p* < 0.001) both decreased from baseline to discharge. Within the EQ-5D-5 L, usual activities (*r* = 0.284, adjusted *p* < 0.001), anxiety/depression (*r* = 0.274, adjusted *p* = 0.005), mobility (*r* = 0.234, adjusted *p* = 0.009), and the VAS health score (*r* = 0.191, adjusted *p* = 0.028) were all statistically significant. Pain/discomfort (*r* = 0.154, adjusted *p* = 0.057) and self-care (*r* = 0.106, adjusted *p* = 0.116) did not reach significance after adjustments, indicating these domains were less responsive or reflected persistent symptoms.

**Table 1:** Comparison of baseline and endline outcomes following multidisciplinary rehabilitation

Outcome Domain	Outcome Measure	Statistical Test	Effect Size	Adjusted *p*-value
Physical & functional	STS	Wilcoxon signed-rank	* r=0.590*	< 0.001
	WSAS (Functional impairment)	Paired t-test	* d=-0.323*	< 0.001
Symptom severity	MRC Dyspnoea scale	Wilcoxon signed-rank	* r=0.491*	< 0.001
	FSS (Fatigue)	Wilcoxon signed-rank	* r=0.382*	< 0.001
Mental health	PHQ-2 (Depression)	Wilcoxon signed-rank	* r=0.321*	< 0.001
	GAD-2 (Anxiety)	Wilcoxon signed-rank	* r=0.314*	< 0.001
QoL (EQ-5D)	Usual Activities	Wilcoxon signed-rank	* r=0.284*	< 0.001
	Anxiety/Depression	Wilcoxon signed-rank	* r=0.274*	0.005
	Mobility	Wilcoxon signed-rank	* r=0.234*	0.009
	Health Score (VAS)	Wilcoxon signed-rank	* r=0.191*	0.028
	Pain/Discomfort	Wilcoxon signed-rank	* r=0.154*	0.057
	Self-care	Wilcoxon signed-rank	* r=0.106*	0.116

Paired t-tests used for normally distributed data (Cohen’s *d*); Wilcoxon signed-rank tests for non-normal data (rank-biserial *r*). p-values adjusted using the Benjamini–Hochberg FDR procedure. *WSAS *Work and Social Adjustment Scale, *FSS *Fatigue Severity Scale, *MRC* (Medical Research Council) Dyspnoea Scale, *PHQ-2* Patient Health Questionnaire-2, *GAD-2* Generalised Anxiety Disorder-2, *QoL (EQ-5D)* Quality of life (EuroQOL Five Dimensions Questionnaire), *VAS* Visual Analogue Scale, *STS-1* (1-minute Sit-to-Stand test)

### Proportion of patients achieving MCID across outcome measures

MCID analysis showed significant improvements across domains, with varying proportions of patients meeting thresholds (Table 2). Physical capacity showed the highest improvement, with 63.5% meeting the MCID on the STS-1 test (*n* = 108; 95% CI: 56.1–70.4%), indicating physical endurance as the most responsive domain to rehabilitation. Breathlessness (MRC) was second at 48.8% achieving MCID (*n* = 83; 95% CI: 41.4–56.3%), followed by depressive symptoms (PHQ-2: 45.9%, *n* = 78; 95% CI: 38.6–53.4%), functional impairment (WSAS: 43.5%, *n* = 74; 95% CI: 36.3–51.0%), and anxiety (GAD-2: 41.8%, *n* = 71; 95% CI: 34.6–49.3%). HRQoL domains showed modest changes. The EQ-5D-5 L score and anxiety/depression reached MCID in 38.2% of participants, while usual activities (36.5%), mobility (33.5%), pain/discomfort (30.6%), and fatigue (27.1%) showed lower rates. Self-care had the lowest rate, with 22.4% meeting MCID (*n* = 38; 95% CI: 16.7–29.2%).

**Table 2:** Proportion of participants achieving the minimal clinically important difference for each outcome measure

Outcome measure	Improved (*n*)	Proportion (%)	95% CI
WSAS	74	43.5	36.3–51.0
FSS	46	27.1	20.9–34.2
MRC	83	48.8	41.4–56.3
PHQ-2	78	45.9	38.6–53.4
GAD-2	71	41.8	34.6–49.3
EQ-5D Mobility	57	33.5	26.9–40.9
EQ-5D Self-care	38	22.4	16.7–29.2
EQ-5D Usual Activities	62	36.5	29.6–43.9
EQ-5D Pain/Discomfort	52	30.6	24.2–37.9
EQ-5D Anxiety/Depression	65	38.2	31.3–45.7
EQ-5D Health Score	65	38.2	31.3–45.7
STS 1-min	108	63.5	56.1–70.4

*WSAS* Work and Social Adjustment Scale, *FSS* Fatigue Severity Scale, *MRC* Medical Research Council Dyspnoea Scale, *PHQ-2* Patient Health Questionnaire-2, *GAD-2* Generalised Anxiety Disorder-2, *EQ-5D-5 L* EuroQol Five-Dimension instrument, *STS 1-min* 1-minute Sit-to-Stand test

These findings show that although the rehabilitation programme was associated with meaningful gains in physical and functional areas for many patients, improvements in QoL and symptom-specific outcomes, such as fatigue and self-care, were less consistent.

### Linear association between referral-to-assessment time and outcome changes

No significant linear association was observed between referral-to-assessment time and the magnitude of change across any outcome measure (*p* > 0.05 for all; Table 3). Correlation coefficients were uniformly small, ranging from − 0.134 to 0.092. For most symptom and functional outcomes — including WSAS (*r* = − 0.105, *p* = 0.172), FSS (*r* = − 0.126, *p* = 0.101), and EQ-5D-5 L self-care (*r* = − 0.134, *p* = 0.081) — negative correlation coefficients suggested greater improvement with longer wait times, though none reached significance. PHQ-2 (*r* = 0.092) and MRC (*r* = 0.028) showed negligible positive correlations.

**Table 3:** Correlation between referral-to-assessment time (days) and change scores for patient-reported and functional outcome measures

Outcome measure (change)	Correlation ($\:\boldsymbol{r}$)	*P*-value
WSAS (Functional Impairment)	-0.105	0.172
FSS (Fatigue)	-0.126	0.101
MRC (Breathlessness)	0.028	0.712
PHQ-2 (Depression)	0.092	0.234
GAD-2 (Anxiety)	-0.017	0.823
EQ-5D-5 L Mobility	0.060	0.435
EQ-5D-5 L Self-care	-0.134	0.081
EQ-5D-5 L Usual Activities	0.050	0.518
EQ-5D-5 L Pain/Discomfort	-0.037	0.636
EQ-5D-5 L Anxiety/Depression	0.002	0.984
EQ-5D-5 L Health Score	-0.075	0.329
STS 1-min (Physical Capacity)	-0.015	0.850

*WSAS* Work and Social Adjustment Scale, *FSS* Fatigue Severity Scale, *MRC* Medical Research Council Dyspnoea Scale, *PHQ-2* Patient Health Questionnaire-2, *GAD-2* Generalised Anxiety Disorder-2, *EQ-5D-5 L* EuroQol Five-Dimension instrument, *STS 1-min* 1-minute Sit-to-Stand test

The linear associations between referral delay and changes in Anxiety (GAD-2: *r* = − 0.017) and physical capacity (STS-1: *r* = − 0.015) were negligible. These results suggest that while no simple linear relationship exists between referral timing and clinical change, the exploratory non-linear analysis reveals a more complex, time-sensitive association between referral delay and patient outcomes.

### Non-linear associations between referral-to-assessment time and outcomes

**Fig. 3:**
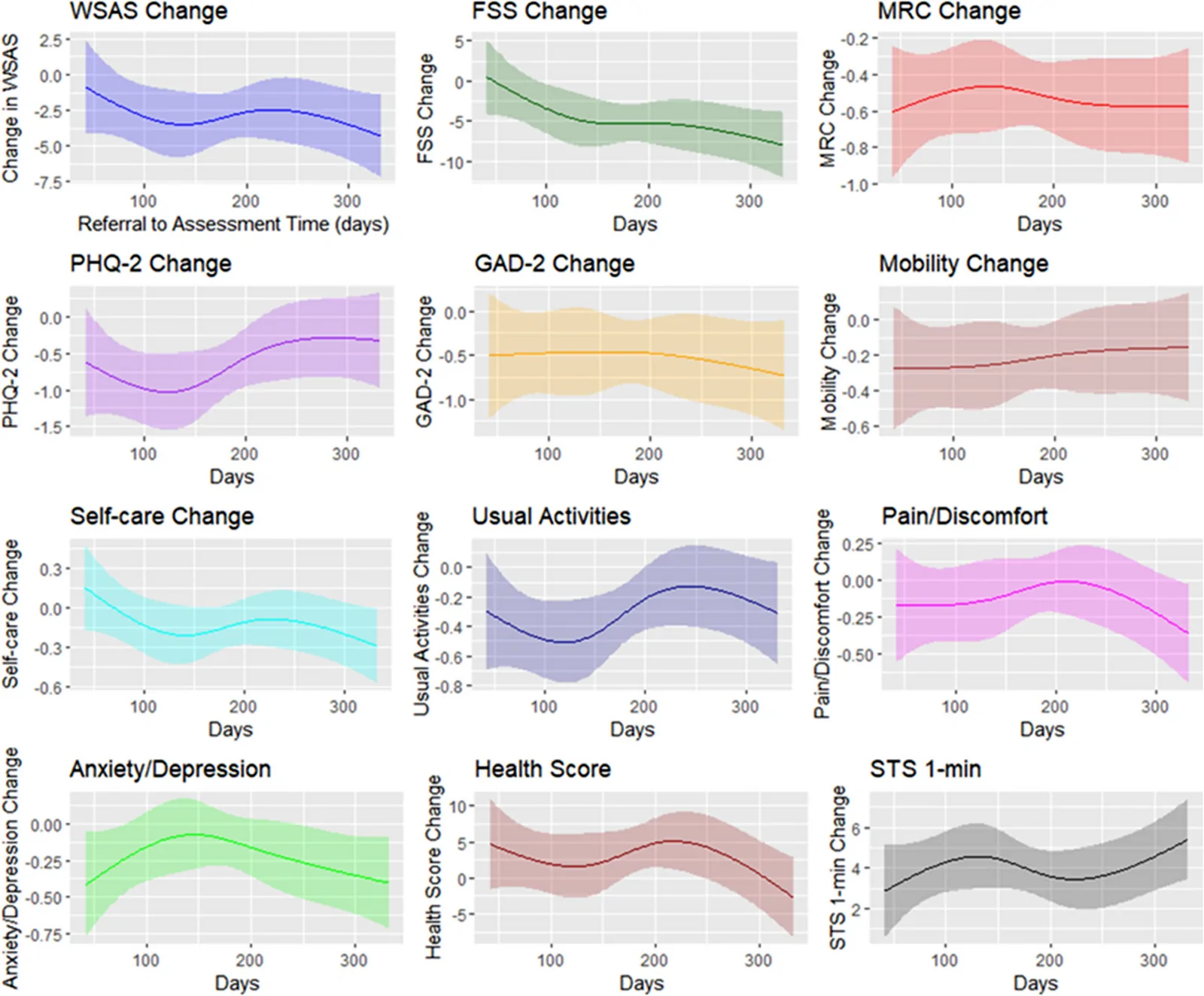
Non-linear associations between referral-to-assessment time and rehabilitation outcome changes. Restricted cubic spline (RCS) models display the relationship between the number of days from referral to first assessment and the change in clinical scores (Endline minus Baseline). Solid lines represent estimated mean change; shaded regions indicate 95% confidence intervals

RCS modelling showed non-linear associations between referral delay and outcome change, not detected by linear correlation (Fig. 3). Functional impairment (WSAS) and depression (PHQ-2) followed a U-shaped trajectory: the greatest magnitude of improvement — reflected in the most negative change scores — was observed in patients assessed between 100 and 150 days after referral. Beyond this window, improvement declined progressively, with predicted change scores trending back towards zero for delays exceeding 200 days. A similar pattern was observed in the EQ-5D-5 L usual activities domain. Fatigue (FSS) and physical capacity (STS-1) followed divergent trajectories. The FSS spline trended downward with increasing delay, indicating progressively greater reductions in fatigue with longer waits, while the STS-1 curve rose markedly beyond 200 days. These patterns may reflect natural recovery or survivorship bias—patients who remain in rehabilitation after delays might be a healthier subgroup, not necessarily due to the delay. Conversely, breathlessness (MRC) and anxiety (GAD-2) showed flat splines with narrow confidence intervals across referral times, suggesting that the magnitude of observed improvement for these outcomes was consistent regardless of assessment timing. Confidence Intervals widened at both extremes of the x-axis (< 50 and > 300 days) due to fewer patients at very short or long delays and greater statistical uncertainty.

## Discussion

This retrospective cohort study assessed the real-world outcomes associated with a structured multidisciplinary rehabilitation programme for Long COVID among 170 patients at a secondary care centre in South London. Three main findings emerge. First, rehabilitation led to significant, meaningful improvements in functional, physical, psychological, and HRQoL domains, especially in physical endurance and breathlessness. Second, MCID confirmed many patients achieved meaningful gains, mainly in physical capacity and mental health, though fatigue, self-care, and pain were less responsive. Third, no clear linear link between referral delay and outcome was found; RCS modelling showed non-linear patterns, hinting at a therapeutic window for improvements, which decreased after about 150 days.

The significant improvement in physical endurance, shown by the largest effect size in the STS-1 test, indicates that deconditioning — not permanent organ damage — mainly causes physical limitations in Long COVID and can be addressed through structured intervention. This interpretation aligns with evidence linking autonomic dysfunction, skeletal muscle mitochondrial impairment, and post-exertional energy dysregulation as reversible causes of functional decline [4, 29]. The substantial improvement in breathlessness supports respiratory physiotherapy and graded activity in multidisciplinary care, aligning with Gloëckl et al.’s findings of improved exercise capacity and dyspnoea after pulmonary rehabilitation in post-COVID patients [10]. Similarly, the significant improvement in functional impairment (WSAS) aligns with findings from Humphreys et al., who identified structured activity engagement as a key driver of occupational reintegration in Long COVID [11]. The more modest response to fatigue (FSS), despite statistical significance, is consistent with the broader literature on post-viral fatigue syndromes, in which fatigue frequently persists beyond the period of structured intervention and may require adjunctive pharmacological, nutritional, or cognitive-behavioural approaches [30]. Only 27.1% of patients met the FSS MCID, suggesting current rehabilitation models may need fatigue-specific strategies beyond graded activity and pacing. While the overall cohort demonstrated significant improvements, the inherent heterogeneity of Long COVID necessitates a cautious interpretation. A critical consideration in this population is the presence of PEM, a hallmark feature where physical, cognitive, or emotional exertion triggers a disproportionate worsening of symptoms [9, 30]. For the PEM-predominant phenotype, traditional aerobic exercise models—originally extrapolated from other post-viral conditions—may be contraindicated and could potentially lead to clinical relapse. Our findings, particularly the more modest response observed in the fatigue domain, underscore the need for rehabilitation to transition from standardised, time-based protocols toward symptom-contingent pacing. This approach prioritises energy conservation and ensures that functional gains in endurance do not occur at the expense of metabolic stability.

The moderate but statistically significant improvements in depression and anxiety are notable, given the high prevalence of psychological comorbidity in Long COVID, estimated at 20–40% across cohort studies [5, 31]. These gains likely reflect an indirect pathway: improved physical capacity and re-engagement with daily activities may have driven psychological recovery, rather than direct psychological intervention, a mechanism previously described in cardiac rehabilitation literature [32]. The failure of pain/discomfort and self-care domains to meet thresholds aligns with evidence that these dimensions represent entrenched somatic symptoms, possibly due to central sensitisation and altered pain processing, that are less responsive to standard exercise rehabilitation [33, 34]. The lack of a clear link between delay and outcome suggests that rehabilitation benefits aren’t solely a function of timing. This is partly reassuring in resource-limited healthcare settings where waiting times are common. However, non-linear RCS modelling offers a nuanced view: a potential therapeutic window exists for functional and psychological domains, with the greatest improvement in patients evaluated within 100–150 days of referral and diminishing returns beyond that. This pattern aligns with the principle that neuroinflammatory and autonomic dysregulatory changes in Long COVID may become increasingly entrenched with prolonged symptom duration, reducing the modifiability of symptoms over time [15, 35]. The upward STS-1 trajectory at longer delays likely reflects survivorship bias—patients remaining after six or more months may be more resilient, not a benefit of late assessment. These findings are the first to provide a non-linear analysis of referral delay effects in a structured Long COVID rehabilitation cohort using validated tools.

### Strengths and limitations

A key strength of this study is the use of established MCID thresholds across a wide range of validated instruments. By moving beyond simple statistical significance (*p*-values), the MCID analysis confirms that the improvements observed in physical capacity, breathlessness, and depression represent tangible, clinically meaningful gains for a substantial proportion of participants. However, the study is limited by the absence of a control group, which limits conclusions: improvements from baseline to discharge can’t be attributed solely to rehabilitation, as natural recovery and regression to the mean may also contribute. However, consistent improvements across domains, effect sizes, and alignment with controlled trials support validity. Furthermore, this study is subject to significant “completer” or “survivorship” bias. The analysis was based solely on the 170 participants who completed the full 12-week programme and both assessments, representing a subset of the 673 distinct patients referred. It is probable that these individuals were the most motivated, possessed higher health literacy, or had symptom profiles that allowed for consistent engagement. Therefore, these results should not be overgeneralised as a definitive measure of programme-observed improvement for the entire Long COVID population, as the outcomes for those who discontinued the programme or were lost to follow-up remain unknown. Accordingly, we describe these findings as “associated with” clinical improvements rather than as evidence of definitive causal effectiveness. Reliance on self-reported outcomes (WSAS, FSS, PHQ-2, and EQ-5D-5L) risks response bias, especially in populations with fluctuating symptoms. Key confounders like vaccination status, COVID-19 severity, comorbidities, and treatments weren’t systematically recorded, and referral delay analysis may be biased by differences between early and late referrers. A single-centre design limits generalisability. Despite these limitations, the use of a large clinical cohort, validated multidomain instruments, and the analysis of temporal effects support the robustness of the core findings.

### Clinical implications and future research

These findings have direct implications for clinical practice and health policy, providing evidence for the funding and structuring of multidisciplinary Long COVID rehabilitation within national care pathways. For physiotherapists and exercise physiologists, the results emphasise that while graded physical activity and respiratory physiotherapy yield consistent gains in physical endurance and breathlessness, these interventions should be integrated with pacing and autonomic conditioning to manage post-exertional malaise effectively. The optimal modality of rehabilitation for Long COVID remains a subject of intense debate within the clinical community. The shift from GET toward ‘pacing’ and ‘autonomic conditioning’ represents an evolution in our understanding of the condition’s pathophysiology. Our multidisciplinary model reflects this transition by integrating physiotherapy with occupational therapy-led pacing strategies. This individualised, symptom-contingent approach aims to provide a safe middle ground: allowing for gradual reconditioning in patients who tolerate it, while enforcing strict activity limits for those with significant autonomic dysregulation or PEM. Clinicians should, therefore, employ rigorous screening to distinguish between these phenotypes, as the ‘one-size-fits-all’ exercise prescription is no longer supported by the emerging evidence base.

Observed clinical outcomes suggest that while graded physical activity and respiratory physiotherapy are associated with consistent, significant gains in physical endurance and breathlessness, persistent issues with pain management and self-care indicate that protocols should be supplemented with targeted pain strategies, occupational therapy, and self-management education for multisystem patients. Furthermore, the identification of a non-linear therapeutic window suggests that services should prioritise early triage and referral within three to five months to maximise recovery potential, although meaningful improvements remain achievable for those with later presentations.

Future care models should continue to refine these distinctions through randomised controlled trials with long-term follow-up. Investigations should specifically focus on phenotype-specific rehabilitation protocols, distinguishing between patients with predominant postural tachycardia syndrome (PoTS), respiratory dysfunction, or cognitive impairment, and examine the safety and efficacy of low-intensity resistance training versus aerobic conditioning across these distinct symptom clusters. Finally, further prospective and controlled studies incorporating formal economic evaluations are needed to evaluate long-term sustainability and the cost-effectiveness of multidisciplinary care models.

## Conclusions

In conclusion, this study evaluated the clinical and patient-reported outcomes of structured multidisciplinary Long COVID rehabilitation and determined whether referral-to-assessment delays affected clinical outcomes. Across functional, physical, psychological, and health-related QoL domains, rehabilitation was associated with statistically significant improvements in most outcome measures, with the greatest gains in physical endurance, breathlessness, and mental health. Fatigue, pain, and self-care proved less responsive, indicating that current rehabilitation models do not yet fully address the heterogeneous symptom burden of Long COVID. Exploratory non-linear modelling suggested that the magnitude of functional and psychological improvement may fluctuate depending on the timing of the assessment. Specifically, these findings indicate a potential therapeutic window, as gains appeared to diminish when referral-to-assessment times exceeded approximately five months. These findings support multidisciplinary Long COVID rehabilitation within national care pathways and highlight the need for targeted interventions for patients with refractory fatigue, pain, and self-care issues.

## Supplementary Information

Supplementary Material 1.

## Data Availability

Data will be made available on request. For data requests, kindly contact the corresponding author.

## Abbreviations

d: Cohen’s *d* (effect size for normally distributed paired data)
EQ-5D-5L: EuroQol Five-Dimension Five-Level instrument
FDR: False Discovery Rate
FSS: Fatigue Severity Scale
GAD-2: Generalised Anxiety Disorder-2
HRQoL: Health-Related Quality of Life
IQR: Interquartile Range
MCID: Minimal Clinically Important Difference
ME/CFS: Myalgic Encephalomyelitis / Chronic Fatigue Syndrome
MRC: Medical Research Council Dyspnoea Scale
NHS: National Health Service
NICE: National Institute for Health and Care Excellence
ONS: Office for National Statistics
PCN: Primary Care Network
PHQ-2: Patient Health Questionnaire-2
r: Rank-Biserial correlation (effect size for non-parametric data)
RCS: Restricted Cubic Spline
r_rb_: Rank-Biserial Correlation coefficient
SD: Standard Deviation
STS-1: 1-minute Sit-to-Stand Test
UK: United Kingdom
VAS: Visual Analogue Scale
WSAS: Work and Social Adjustment Scale
