# Supplementary material for: Real-world outcomes of a multidisciplinary rehabilitation programme for adults with long COVID: a retrospective cohort study
Source: BMC Public Health. 2026 Aug 13;26:3302. doi: 10.1186/s12889-026-29023-3 (PMC13749804; doi:10.1186/s12889-026-29023-3)
Supplement: Supplementary file 1 [file 12889_2026_29023_MOESM1_ESM.docx]

Supplementary File 1: Summary of variables and outcome measures in the data

| Variable category | Variable / Instrument | Description and scoring |
| --- | --- | --- |
|  | Referral-to-Assessment Time | Calculated as the number of days between the date of referral and the date of the first rehabilitation session. |
| Functional Outcomes | Work and Social Adjustment Scale (WSAS) | A 5-item measure assessing symptom impact on work, home management, social activities, private leisure, and relationships.  Range: 0–40^1^. |
|  | 1-minute Sit-to-Stand (STS-1) | Measures lower limb strength and endurance by counting the number of full stands from a seated position in one minute^2^.  Range: Count of repetitions. |
| Symptom Severity | Fatigue Severity Scale (FSS) | A 9-item scale assessing the impact of fatigue on daily living.  Range: 1–7 (mean of items)^3^. |
|  | MRC Dyspnoea Scale | A 5-point scale evaluating breathlessness severity during activity.  Range: 1–5^4^. |
| Psychological Outcomes | Patient Health Questionnaire-2 (PHQ-2) | A 2-item screening tool for depression symptoms over the past two weeks^5^.  Range: 0–6. |
|  | Generalised Anxiety Disorder-2 (GAD-2) | A 2-item screening tool for anxiety symptoms ^6^.  Range: 0–6 |
| Quality of Life | EQ-5D-5L (Domains) | Assesses health-related quality of life across five domains: Mobility, Self-care, Usual activities, Pain/discomfort, Anxiety/depression.  Range: 1–5 per domain. |
|  | EQ-5D Visual Analogue Scale (VAS) | Self-rated overall health status on a vertical visual^7,8^.  Range: 0–100. |

References

1. J.C. Mundt, I.M. Marks, M.K. Shear, J.H. Greist. The Work and Social Adjustment Scale: a simple measure of impairment in functioning. Br J Psychiatry, 180 (2002), pp. 461–464, 10.1192/bjp.180.5.461
2. R.W. Bohannon Hand-grip dynamometry predicts future outcomes in ageing adults J Geriatr Phys Ther, 31 (2008), pp. 3–10, 10.1519/00139143-200831010-00002
3. L.B. Krupp, N.G. LaRocca, J. Muir-Nash, A.D. Steinberg. The fatigue severity scale. Application to patients with multiple sclerosis and systemic lupus erythematosus Arch Neurol, 46 (1989), pp. 1121–1123, 10.1001/archneur.1989.00520460115022
4. J.C. Bestall, E.A. Paul, R. Garrod, R. Garnham, P.W. Jones, J.A. Wedzicha. Usefulness of the Medical Research Council (MRC) dyspnoea scale as a measure of disability in patients with chronic obstructive pulmonary disease. Thorax, 54 (1999), pp. 581–586, 10.1136/thx.54.7.581
5. K. Kroenke, R.L. Spitzer, J.B.W. Williams. The Patient Health Questionnaire-2: validity of a two-item depression screener. Med Care, 41 (2003), pp. 1284–1292, 10.1097/01.MLR.0000093487.78664.3C
6. A. Sapra, P. Bhandari, S. Sharma, T. Chanpura, L. Lopp L. Using Generalized Anxiety Disorder-2 (GAD-2) and GAD-7 in a Primary Care Setting. Cureus. 2020 May 21;12(5):e8224. doi: 10.7759/cureus.8224. PMID: 32582485; PMCID: PMC7306644.
7. M. Herdman, C. Gudex, A. Lloyd, et al. Development and preliminary testing of the new five-level version of EQ-5D (EQ-5D-5L) Qual Life Res, 20 (2011), pp. 1727–1736, 10.1007/s11136-011-9903-x
8. Y. Feng, D. Parkin, N.J. Devlin. Assessing the performance of the EQ-VAS in the NHS PROMs programme. Qual Life Res. 2014 Apr;23(3):977-89. doi: 10.1007/s11136-013-0537-z.

Supplementary File 2. Longitudinal review of referrals to the long COVID rehab service (2021–2023). (a) The total referrals by calendar year reveal a steep drop after the 2021 peak. (b) Referral data broken down by age group and year, emphasising the dominance of the 45–64 age range. (c) Referrals sorted by sex and year, showing a steady majority of females. (d) Referral data categorised by ethnicity and year. (*N*=1,269)
